# Effect of intraocular pressure on crystalline lens oscillations: a computational study using porcine eye model

**DOI:** 10.1371/journal.pone.0320205

**Published:** 2025-03-25

**Authors:** Ali Dahaghin, Milad Salimibani, Agnieszka Boszczyk, Jorge Grasa, Damian Siedlecki

**Affiliations:** 1 Department of Optics and Photonics, Wroclaw University of Science and Technology, Wrocław, Poland; 2 Aragón Institute of Engineering Research (I3A), University of Zaragoza, Zaragoza, Spain; Bascom Palmer Eye Institute, UNITED STATES OF AMERICA

## Abstract

This study addresses a crucial knowledge gap by investigating the impact of intraocular pressure (IOP) on the wobbling characteristics of the crystalline lens in an ex vivo setting. It utilizes previous validated computational porcine eye models, which offer anatomical and physiological similarities to the human eye. These models incorporate fluid-structure interaction (FSI) to simulate the mechanical interaction between the fluids of the eye and the solid structures. Simulations were conducted under constant mechanical properties and boundary conditions, allowing for precise quantification of lens wobbling behavior with varying IOP levels. Various trends in lens displacement were observed at various IOP levels, revealing significant variations in both magnitude and duration. The results demonstrate the central role of intraocular pressure in influencing lens overshooting during rotational motion, with potential clinical implications. The observed lens displacement patterns, particularly in conditions like glaucoma, underscore the importance of considering IOP as a critical factor in understanding ocular biomechanics. Beyond immediate biomechanical relevance, the study’s findings suggest the potential use of the Purkinje imaging system as a non-invasive method for IOP estimation based on lens overshoot as an “inverse solution” strategy. This non-invasive imaging technique offers a promising alternative to traditional methods, minimizing patient discomfort and potentially enhancing measurement precision.

## 1. Introduction

The crystalline lens, a transparent structure with biconvex geometry, serves as a critical element in the optical system of the eye [1]. The biomechanical properties of the lens and other structures have been found to be influenced by various factors, with intraocular pressure (IOP) being one of them. Previous research suggests that changes in IOP can affect the mechanical behavior of the eye [2,3]. The fluid pressure inside the eye is known as IOP and fluctuations in it have been linked to a number of ocular conditions, particularly glaucoma [4]. These fluctuations can have significant effects on the behavior of the lens, affecting the retina’s capacity to focus light efficiently. It may also prevent the lens ability from making proper accommodation, resulting in difficulties in focusing on objects at different distances. Large fluctuations might result in the lens shifting inside the eye, altering the normal path of light and causing distorted or blurred vision [5–9].

The crystalline lens, a flexible structure, dynamically adapts its curvature and thickness to accommodate near and far vision [10]. In addition, during gaze shifts, which occurs when the gaze is rapidly shifted from one point to another, the eye undergoes rapid rotational motions. Right after the eyeball stops moving, the lens wobbles for a moment before returning to its natural position. Crystalline lens wobbling is a natural phenomenon that typically lasts only milliseconds. This concept refers to the oscillatory movements of the lens, which can occur during eye rotations as impacts of inertial forces and can be visualized using Purkinje images, reflections of light sources that the lens surfaces have created [11]. Understanding this phenomenon is critical because it could potentially have some effects on vision. To maintain an optimal refractive function, the position and stability of the crystalline lens within the ocular environment are extremely important. However, factors like IOP, can disrupt this delicate balance. Nevertheless, there has not been much research done on the specific effects of IOP on the dynamics of lens movement.

Biomechanical models employing the finite element method (FEM) provide helpful techniques and tools to simulate complex systems. In the context of eye modeling, FEM can take into account variables that include IOP, geometry, and properties, to provide a quantitative characterization of the biomechanical environment in the field of ophthalmology [12–14]. Salimi et al. suggest that variations of IOP can affect the natural frequencies of the lens and its vibrational characteristics [15].

This study aims to bridge the knowledge gap by exploring the influence of IOP on the wobbling characteristics of the crystalline lens in an ex vivo setting. Pig eyes are anatomically and physiologically comparable to human eyes [16]; using them as a model will have several advantages for future studies [17]. The investigation involves specific changes in IOP within a validated porcine eye model coupled with advanced fluid-structure interaction (FSI) techniques to quantify the lens wobbling under parameters that are controlled. Through this research, the findings may not only have direct clinical relevance, such as a novel approach to measuring intraocular pressure, but also contribute to the general field of ocular biomechanics.

## 2. Materials and methods

**Fig 1:**
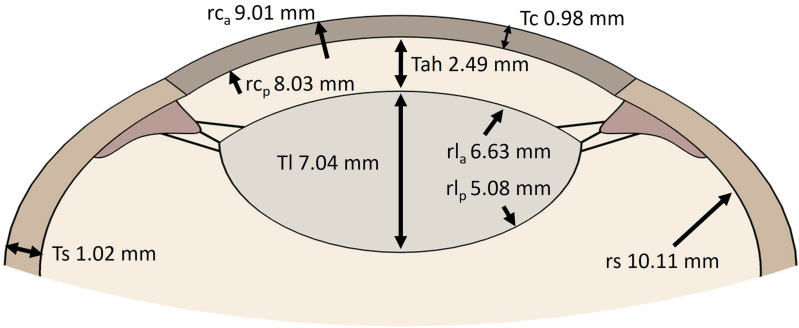
Principal dimensions considered for the geometry of the porcine eye.

In the present study, a validated optomechanical eye model was used to analyze the mechanical behavior of a porcine eye under different IOP levels [18]. Optomechanical eye models of porcine eyes were developed in COMSOL Multiphysics. They incorporated fluid-structure interaction to emulate the mechanical interplay between the humors and the solid structures of the eye. These comprehensive models encompass essential ocular components, such as the crystalline lens, cornea, sclera, lens, zonules (segmented into anterior, equatorial, and posterior regions), ciliary muscle, vitreous body, and aqueous humor (Fig 1).

Geometric values were sourced from existing literature [19,20]. The total length of the eyeball was 22.94 millimeters. The anterior (rc_a_) and posterior (rc_p_) radius of the cornea were 9.01 and 8.03 mm, respectively, with a thickness (Tc) of 0.09 mm. The lens exhibits a central thickness (Tl) measuring 7.04 mm, along with different radii for its anterior (rl_a_) and posterior (rl_p_) surfaces, quantified at 6.63 and 5.08 mm. It was conceptualized as being suspended by three pairs of zonular fibers, each with a uniform width of 50 µm [21,22]. The thickness of the sclera (Ts) was adjusted to be 1.02 millimeters, accompanied by a radius (rs) of 10.11 millimeters. The inclusion of the choroid and retina in rotational motion simulations was deemed unnecessary, primarily because of its minimal thickness. Consequently, these components were left out of the modeling process. The considered anatomical parameter, denoting the dimensions of the porcine eye, plays a crucial role in ocular biomechanics and can have implications for various studies.

Vitreous body and aqueous humor represented as liquids in the model; Both as a viscous Newtonian, incompressible fluid. Dynamic viscosity was assigned a value of 0.00074 Pa ∙ s, and a density of 1000 kg/m^3^ was applied to accurately assume the dynamic behavior of the ocular fluids [23]. These components subjected to varying pressure conditions played the role of determining the IOP magnitude in the model. A range of diverse IOP values, specifically varying from 15 to 20 mmHg (totaling 6 values) were systematically applied to the inner surface of the eye globe. Although each model was subjected to different IOP magnitudes, the material properties and the boundary conditions were kept the same for solid parts. Table 1 shows the mechanical properties of the different tissues of the eye model.

**Table 1:** Mechanical properties of the solid tissues [23,24].

Sclera	*E*^1^ = 28 MPa, υ^2^ = 0.49, *ρ*^3^ = 1400 kg/m^3^
Cornea	*E* = 12 MPa, υ = 0.48, *ρ*=1400 kg/m^3^
Ciliary Muscle	*E* = 11 MPa, υ = 0.45, *ρ*=1600 kg/m^3^
Zonule fibres	*E* = 0.95 MPa, υ = 0.49, *ρ*=1000 kg/m^3^
Crystalline lens	*E* = 1.5 MPa, υ = 0.49, *ρ*=1100 kg/m^3^

^1^Young’s modulus,

^2^Poisson’s ratio,

^3^Density

Triangular elements were used to represent irregular geometry in all domains, resulting in a model with 48,139 elements. Identification of the optimal mesh size ensued from a sensitivity analysis carried out within the computational model. It has an average quality of 0.82, based on a built-in quality assessment. This assessment yields a quantitative rating within the range of 0 to 1, facilitating the evaluation of the quality of the elements [25].

**Fig 2:**
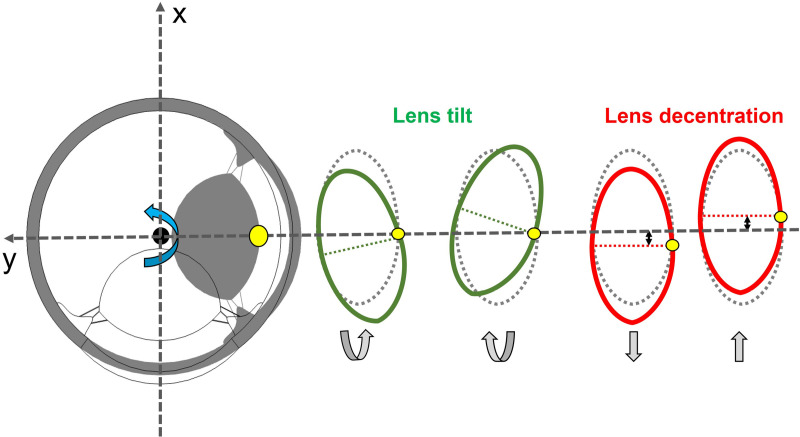
Illustration of decentration and tilt of the crystalline lens being induced by rotation motion of the whole eye globe. Lens decentration refers to the misalignment of the optical center of the lens with the center of the lens mount. Lens tilt refers to the angular misalignment of the lens relative to the optical axis of the eye globe.

In the realm of computational biomechanics, the accuracy and efficiency of simulations hinge greatly on the precise setting of governing equations and boundary conditions. These settings were employed from the previous study [18]. The central region of the sclera, functioning as the center of rotation of the eyeball, maintains a fixed position without linear movement. The apex point of the crystalline lens, denoted as yellow points in Fig 2, serves as the reference point for capturing the lens’ mechanical displacement (both tilt and decentration of the crystalline lens). These displacements occur in response to inertial movements. The crystalline lens, in response to this movement, exhibits an inertial movement. In this rotational motion, where the eyeball completes a 90 [deg] revolution around its vertical axis, the maximum angular velocity reaches a value of 1700 [deg/s] [18]. For a better understanding of the crystalline lens behavior during this rotation, see the supporting information.

**Fig 3:**
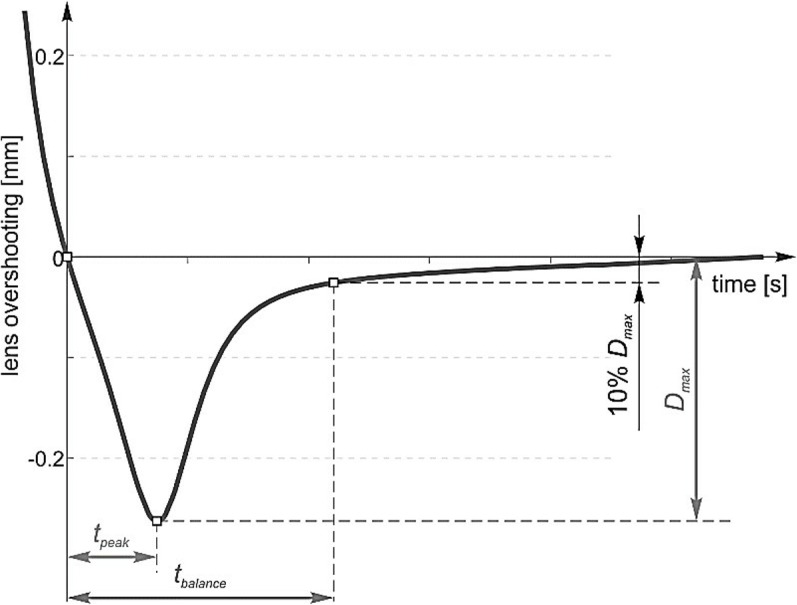
Quantified parameters of the crystalline lens apex displacement.

Furthermore, the quantification of the displacement of the crystalline lens apex involves the incorporation of three numerical indicators, which are marked in Fig 3, namely the time of the appearance of the maximum displacement (*t*_*peak*_), the stabilization time (*t*_*balance*_) and the maximum displacement (*D*_*max*_). The maximum displacement time represents the moment when the crystalline lens undergoes its greatest displacement, while the stabilization time signifies the point in time when the lens recovers to 10% of its overall displacement. Additionally, the maximum displacement is the combination of lens decentration and tilt (Fig 2), quantifies the extent of lens displacement precisely at *t*_*peak*_. These indicators contribute to a comprehensive understanding of the dynamic aspects associated with crystalline lens wobbling.

## 3. Results

The displacement of the lens in the porcine eye was evaluated at different intraocular pressures (IOPs) after a sudden stop after a 90 deg rotation, with the goal of examining how IOP affects lens dynamics. This specific pattern of lens wobbling, referred to as overshooting, was expected because, in ex vivo studies, the assumption is that the lens is in a non-accommodated state. In this state, the lens is stretched by the zonules, making it more stable, and the anticipated pattern of overshooting was observed [26].

**Fig 4:**
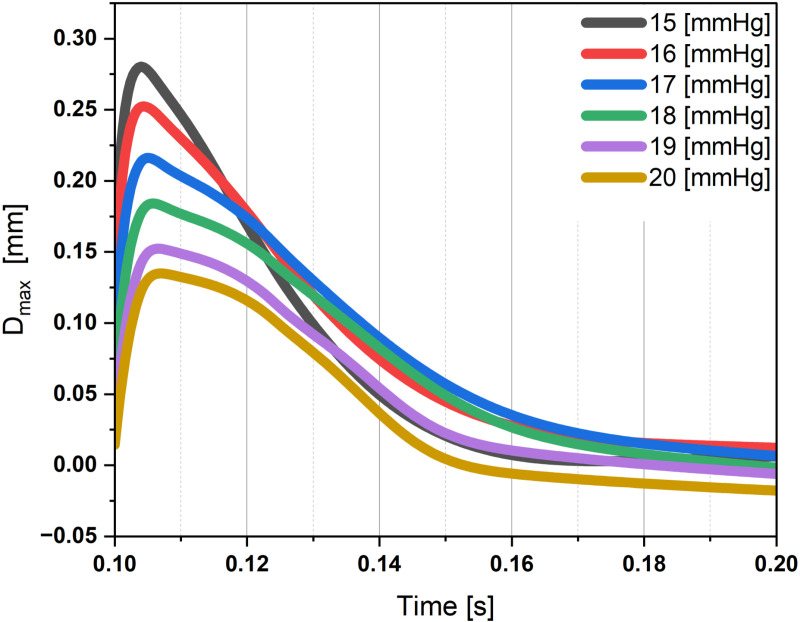
Time evolution of apex position of the crystalline lens during overshooting. It indicates the lens shift from the equilibrium position after the end of the rotation and returning to the initial position.

As seen in Fig 4, the results illustrated a significant inverse correlation between IOP and *D*_*max*_. With increasing IOP, a consistent decline in maximum displacement was observed. For example, at an IOP of 15 mmHg, the lens demonstrated a *D*_*max*_ of 0.280 mm, indicating relatively unimpeded lens movement during sudden stops. Conversely, at an IOP of 20 mmHg, *D*_*max*_ decreased to 0.135 mm, suggesting increased resistance to lens displacement due to higher IOP levels (Table 2).

**Table 2:** The maximum velocity and acceleration data of the apex position of the crystalline lens during overshooting for all IOP levels.

IOP	[mmHg]	15	16	17	18	19	20
Velocity	[µm/ms]	22.2	19.2	13.5	10.4	8.4	7.5
Acceleration	[µm/ms^2^]	56.4	49.6	42.3	37.9	34.6	31.8

The findings indicate that higher levels of IOP limit the lens’s ability to move, leading to a decrease in *D*_*max*_. This phenomenon can be attributed to the mechanical properties of the eye, by which elevated IOP creates increased resistance to lens displacement. As indicated in Table 2, one of the theories for this resistance is the notable impact of IOP on the velocity and acceleration of the apex position of the crystalline lens during overshooting. Both of these factors exhibit an inverse correlation with rising pressure. The restriction in lens movement under conditions of elevated IOP suggests that the eye encounters stronger opposing forces, impeding the lens’s free motion (S1 Fig).

*t*_*peak*_ remained relatively stable across different IOPs, ranging from 0.104 to 0.107 seconds in terms of temporal characteristics. This suggests that the lens consistently reached its *D*_*max*_ within a defined timeframe following the sudden stop, regardless of the applied IOP. In contrast, *t*_*balance*_ showed slight variability across IOPs, ranging from 0.154 to 0.188 seconds. Further research is needed to elucidate the factors that contribute to this minor variation and to unravel the underlying mechanisms governing the temporal dynamics of lens balance.

All detailed representations of variations (percentage of changes [%]) in the measured parameters compared to the standard pressure of 15 mmHg are provided in **Table 3**. These tabulated data provide an overview of the deviations in these parameters and their difference from the established baseline.

**Table 3:** Overshooting parameters for the six IOP levels and the percentage variation with respect to the standard level of 15 [mmHg].

IOP [mmHg]	15	16	17	18	19	20
*t*_*peak*_ [s]	0.104	0.1044	0.1052	0.1056	0.1064	0.1068
		0.4%	1.2%	1.5%	2.3%	2.7%
*t*_*balance*_ [s]	0.154	0.170	0.185	0.188	0.186	0.181
		10.7%	20.3%	22.1%	20.8%	18.0%
*D*_*max*_ [mm]	0.280	0.252	0.216	0.184	0.152	0.135
		-9.9%	-22.9%	-34.3%	-45.7%	-51.9%

**Fig 5:**
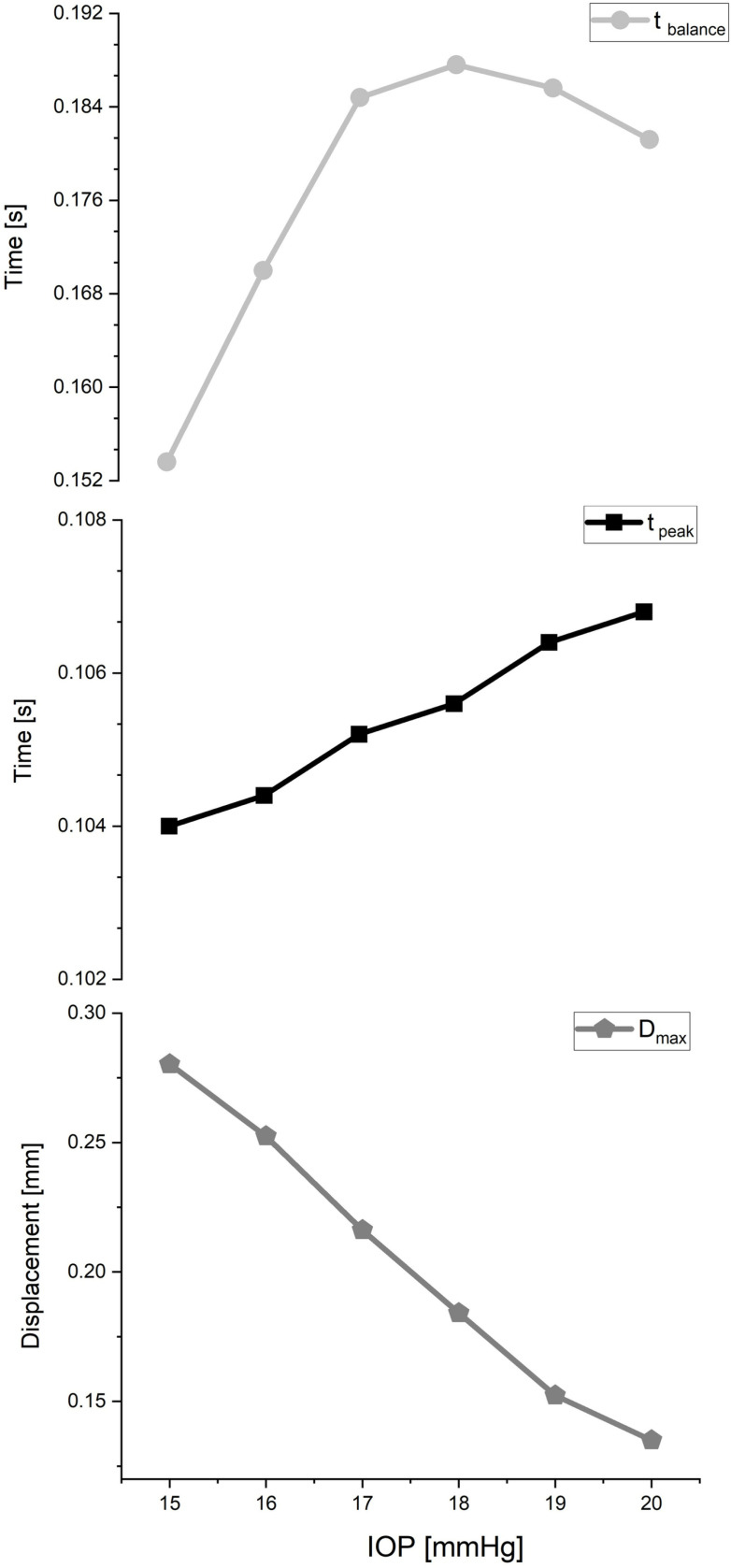
The trend of observed changes in t_balance_, t_peak_ and D_max_, with increasing IOP levels.

The simulations reveal distinct patterns of displacement and time under varying IOP conditions. In particular, all the analyses showcased disparities in both the magnitude and duration of lens displacement across different levels of IOP (Fig 5). These findings underscore the significant influence of intraocular pressure on the mechanical behavior of the lens.

**Fig 6:**
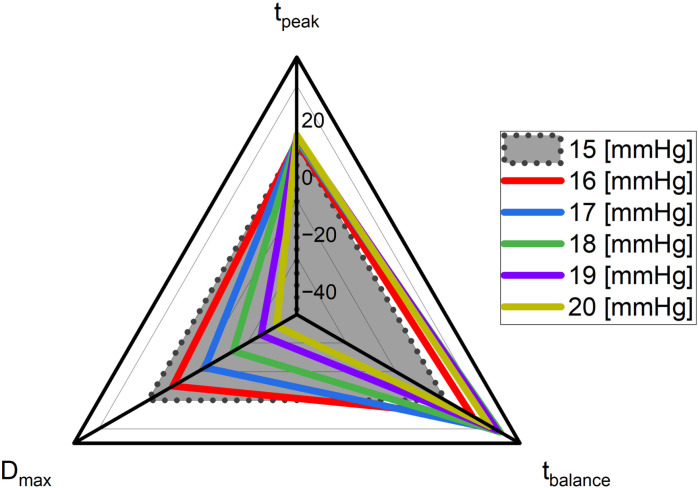
Analyzing trends based on the percentage change in lens displacement patterns across various levels of intraocular pressure.

Through simulations, we identified unique trends in lens displacement patterns at various IOP levels. In particular, the results showed that IOP alterations caused notable disparities in both the extent and duration of lens displacement (Fig 6), underscoring the significant role of intraocular pressure in influencing lens overshooting.

## 4. Discussion

The biomechanical response of the eye to sudden movements can have a significant impact on intraocular pressure (IOP), potentially resulting in vision-threatening consequences [27]. Gaining a complete understanding of the dynamic behavior of the lens, a crucial component of the eye, is vital to identifying potential vulnerabilities and informing clinical practices.

The correlation between IOP and *D*_*max*_, which was found to be inversely related, emphasizes the need to take IOP levels into account when evaluating lens behavior in scenarios involving sudden deceleration. These findings are consistent with previous research indicating that elevated IOP induces stress in the lens capsule [7], resulting in smaller overshoot displacements. The increased damping due to the higher IOP [28] can counteract this, leading to a decrease in overshooting and a small delay in *t*_*peak*_. This discovery highlights the need for further exploration into the specific effects of changes in stiffness and damping on observed dynamics.

The observed inverse relationship between IOP and *D*_*max*_ underscores the importance of considering IOP as a contributing factor when evaluating ocular motion during sudden stops. The findings emphasize that higher levels of IOP restrict the movement of the lens, resulting in a decrease in *D*_*max*_. This implies that an elevated IOP creates greater resistance to lens displacement, hindering its ability to undergo significant movement during sudden deceleration events. These results highlight the role of IOP in influencing conditions associated with elevated IOP, such as glaucoma or ocular trauma [29].

The consistent of the *t*_*peak*_, regardless of IOP levels, indicates a strong reaction of the lens to abrupt stops. This discovery suggests the presence of a finely tuned mechanism that governs the timing of lens displacement during sudden deceleration events. The lens’s ability to reach its *D*_*max*_ consistently within a defined time frame is crucial for maintaining visual stability and minimizing potential damage to ocular structures.

In contrast, the observed variation in *t*_*balance*_, the duration needed for the lens to stabilize after reaching its maximum displacement, suggests that factors beyond intraocular pressure (IOP) play a role in lens stabilization. These may include the elasticity of the lens itself and the biomechanical characteristics of the surrounding ocular structures [30]. The complex interaction between these factors probably causes the observed fluctuations in *t*_*balance*_. The results indicate that elevated IOP affects not only the lens’ initial response, but also extends the time of lens movement after dynamic disturbances. Such findings imply that high IOP might impede the lens ability to return efficiently to its original position following these disturbances. Additionally, the damping effectiveness of these factors could be particularly significant. Therefore, further research is necessary to understand the mechanisms that govern lens stabilization.

The clinical implications of the relationship between intraocular pressure (IOP), lens displacement, and vision are significant. An increase in IOP, in addition to contributing to conditions such as glaucoma, leads to heightened resistance from surrounding eye tissues, resulting in reduced lens displacement. This reduction in lens displacement can affect visual acuity [31,32]. The dynamic nature of IOP throughout the day can cause fluctuations in lens displacement, affecting vision, and leading to changes in accommodation, potentially influencing near vision. Understanding these relationships is crucial for the effective diagnosis and management of ocular conditions, particularly glaucoma, and for forming treatment strategies. Furthermore, the relationship between IOP, lens displacement, and vision is influenced by individual variations in eye anatomy, biomechanics, and tissue properties. Factors such as age, ocular health, and the presence of diseases can affect the lens response to changes in IOP [33–35]. Therefore, future research should focus on gaining a deeper understanding of the biomechanical mechanisms underlying these relationships. This research should also explore diurnal variations in lens dynamics and investigate the long-term effects of IOP fluctuations on lens displacement and visual function. Furthermore, exploring the effects of different dynamic loading conditions, such as varying rotation speeds and magnitudes, could provide valuable insights into the lens’s adaptability to diverse stimuli. Acquiring this knowledge will allow the development of improved diagnostic tools and treatment approaches in the field of ophthalmology.

## 5. Conclusion

The results of this investigation provide valuable insight into how the lens responds to changes in intraocular pressure (IOP) using porcine eyes as models. By conducting finite element analysis, distinct patterns of lens displacement can be observed in response to different levels of IOP. Significant differences in both the magnitude and duration of lens displacement under varying IOP conditions highlight the complex relationship between intraocular pressure and eye biomechanics. These findings emphasize the importance of considering IOP as a critical factor in understanding the mechanical behavior of ocular tissues. The observed variations in lens displacement patterns have important implications for clinical practice and research in ocular biomechanics, particularly in conditions like glaucoma, where elevated IOP is a primary risk factor.

These results contribute to a better understanding of how ocular structures mechanically respond to pressure changes, which can aid in the development of more accurate diagnostic tools and personalized treatment strategies for people with glaucoma and other ocular diseases characterized by dysregulation of IOP. This research can delves into the potential of employing the optical Purkinje imaging system as an “inverse solution” strategy to estimate intraocular pressure (IOP) by leveraging lens overshoot. Although current methods, such as air puffs, offer valuable information, they can potentially cause discomfort for patients. In contrast, the Purkinje imaging system, a non-invasive imaging technique, holds promise in terms of enhancing patient comfort and potentially improving the precision of IOP measurements. Furthermore, our study highlights the importance of incorporating variations in IOP in computational models of ocular biomechanics to improve their predictive precision and clinical relevance.

Although this study has provided valuable information on the interaction between IOP and lens dynamics, it is important to acknowledge certain limitations. The use of an ex vivo model, although validated, may not fully capture the complex intricacies of the eye in vivo. Therefore, caution should be exercised when extrapolating the findings to human eyes. Future studies should incorporate in vivo measurements and analysis of the entire wobbling phenomenon to gain a more holistic understanding of lens dynamics under dynamic loading conditions.

## Supporting information

S1 Fig crystalline lens overshooting animation. (GIF)
