# Supplementary figures and images for: Effect of intraocular pressure on crystalline lens oscillations: a computational study using porcine eye model
Source: PLoS One. 2025 Mar 25;20(3):e0320205. doi: 10.1371/journal.pone.0320205 (PMC11936197; doi:10.1371/journal.pone.0320205)

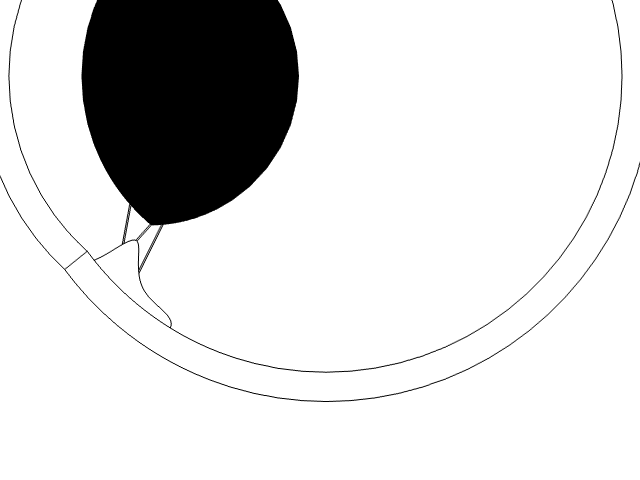

Supplement: S1 Fig — (GIF) [file pone.0320205.s001.gif]
